# Genomic diversity and antimicrobial resistance of *Campylobacter* spp. from humans and livestock in Nigeria

**DOI:** 10.1186/s12929-022-00786-2

**Published:** 2022-01-24

**Authors:** Benshak J. Audu, Strachan Norval, Lopes Bruno, Ramjee Meenakshi, Macrae Marion, Ken J. Forbes

**Affiliations:** 1 National Veterinary Research Institute, Vom, Nigeria; 2 School of Biological Sciences, Cruickshank Building, University of Aberdeen, St. Machar Drive, Aberdeen, UK; 3 School of Medicine, Medical Sciences & Nutrition, University of Aberdeen, Foresterhill, Aberdeen, UK

**Keywords:** *Campylobacter*, Multilocus sequence typing, Clonal complex, Sequence type, Antimicrobial resistance

## Abstract

**Background:**

*Campylobacter* spp. are zoonotic pathogens, ubiquitous and are found naturally as commensals in livestock from where they can be transmitted to humans directly or through animal products. The genomic diversity and antimicrobial resistance profile of *Campylobacter* was investigated with a focus on *C*. *jejuni* and *C*. *coli* in humans and livestock (poultry and cattle) from Nigeria.

**Methods:**

586 human stool samples and 472 faecal samples from livestock were cultured for thermophilic *Campylobacter* species on modified charcoal cefoperazone deoxycholate agar (mCCDA). Culture in combination with whole genome sequencing identified and confirmed the presence of *Campylobacter* in humans and animals from the study area. Further analysis of the sequences was performed to determine multilocus sequence types and genetic determinants of antimicrobial resistance to fluoroquinolone, betalactam, tetracycline and macrolide classes of antimicrobials.

**Results:**

From the human stool samples tested, 50 (9%) were positive of which 33 (66%) were *C*. *jejuni*, 14 (28%) were *C*. *coli* while 3 (6%) were *C*. *hyointestinalis*. In livestock, 132 (28%) were positive. Thirty one (7%) were *C*. *jejuni* while 101 (21%) were *C*. *coli*. Whole genome sequencing and MLST of the isolates revealed a total of 32 sequence types (STs) identified from 47 human isolates while 48 STs were identified in 124 isolates from livestock indicating a population which was overall, genetically diverse with a few more dominant strains. The antimicrobial resistance profiles of the isolates indicated a higher prevalence of resistance in *Campylobacter* isolated from livestock than in humans. Generally, resistance was greatest for betalactams (42%) closely followed by fluoroquinolones (41%), tetracyclines (15%) and lastly macrolides (2%). Multidrug resistance to three or more antimicrobials was observed in 24 (13%) isolates from humans (n = 1, 4%) and chicken (n = 23, 96%).

**Conclusions:**

This study has further contributed information about the epidemiology, genetic diversity and antimicrobial resistance profile of thermophilic *Campylobacter* in Nigeria.

## Background

*Campylobacter* is considered to be the biggest cause of human bacterial gastroenteritis globally. Diarrhoea, one of the most common outcomes of infection, leads to the mortality of about 1.4 million children yearly, the majority occurring in the developing world where poor hygiene as well as contaminated water and foods are more prevalent [1].

*Campylobacter* has been isolated from apparently healthy people as well as children having diarrhoea in developing countries and could be due to early exposure with a subsequent development of protective immunity [2, 3].

In humans, *Campylobacter jejuni* is the main cause of disease though about 10% is by *C. coli* [4]. The reference *Campylobacter jejuni* NCTC11168 genome sequence has a circular chromosome of approximately 1.6 Mb with a G + C content of 30.6%. Predicted coding sequences (CDS) number slightly more than 1,600 with an average gene length of 948 bp and more than 90% of the genome coding for proteins. The genome also contains hypervariable sequences in genes which function to modify surface structures and is thought to aid survival strategy of the pathogen [5].

Studies on the emergence, spread, persistence and evolution of *Campylobacter* species in environmental niches have been carried out using molecular tools to examine genome diversity [6, 7] and how this relates to human and animal health. These molecular and computational tools are used to infer the population structure of the organism and to understand evolutionary relationships within geographical locations and the various hosts [8]. These tools have also enabled researchers to track pathogen movements, to understand their origins and how environmental drivers of disease spread play a role [9, 10]. Due to infrastructural deficits though, these tools, are hardly used to study pathogens in Africa creating a knowledge gap.

*Campylobacter* induced enteritis is usually self-limiting in many individuals and treatment is generally not recommended. However, in cases of extra-intestinal infections, in immunocompromised patients, the elderly and very young, antimicrobial agents such as erythromycin and ciprofloxacin are the drugs of choice [11]. Studies of human infections caused by resistant and sensitive *Campylobacter* strains has shown that ill-health is more severe and prolonged in infection with resistant strains [12, 13]. The growing rate at which resistance to these drugs is developing though, has made treatment harder, more so in poorer countries such as Nigeria. Resistance to ciprofloxacin, tetracycline and erythromycin have been observed to be the most common forms of resistance in *Campylobacter* spp. by various authors mostly employing disc diffusion methods [14].

The level of impact antimicrobial resistant *Campylobacter* of livestock origin has had on human health is still not fully understood, however, the close association between humans and animals in Nigeria will likely make livestock ownership a major risk factor for pathogen and antimicrobial resistance transfer.

Study of *Campylobacter* isolated from animals and humans in Nigeria using whole genome sequence analysis is currently unavailable. That makes this study a first of its kind and aims to characterize, determine genetic diversity and antimicrobial resistance in *Campylobacter* from Nigeria.

## Methods

### Statement of ethical approval and consent to participate

The study of human participants was approved by the Health Research Ethics Committee, Plateau State Specialist Hospital, Plateau State, Nigeria – NHREC /05/01/20106 (Appendix I). An informed consent was also written and signed by all the human subjects in this study or from parents/guardians of minors.

### Isolation of Campylobacter from humans and animals

A total of 568 human stool samples were collected from 248 symptomatic participants described as having diarrhoea, abdominal pain with or without other symptoms such as stomach upsets, fever, vomiting, blood in the stool, who had submitted stool samples to a medical facility as part of their diagnostic requirements. The other 320 samples were collected in schools from asymptomatic participants showing no self-reported clinical symptoms as described above. All the samples were collected in sterile specimen bottles, placed in a cool box with ice, transported and processed within 3 h of collection at the laboratory. Samples were collected from patients aged between 2 months and 89 years.

A simple questionnaire was completed by each of the human participants. The information gathered from each participant through the questionnaire was linked to each isolate, in a spreadsheet and used for subsequent analyses.

Chicken and other poultry faecal samples were collected from live poultry markets, slaughter/processing points and veterinary clinics at post mortem. Three hundred and twelve poultry faecal samples were collected at slaughter (n = 176), at post mortem from veterinary clinics (n = 71), poultry farm (n = 19) and from indigenous Fulani ecotype chicken at rural live bird markets (n = 46). Individual samples were collected immediately after excretion from live birds and directly from the caecum at slaughter or post mortem in sterile bijou bottles, placed in a cooler box and transported to the laboratory for culturing within 3 h post collection.

Cattle faecal samples totalled 160 of which 149 were collected at the abattoir and the remaining eleven at a cattle farm. Samples collected at the abattoir were from animals bought by butchers from herdsmen to be slaughtered and carcasses dressed for subsequent sale to local meat sellers. Most of these animals were not raised on ranches or farms but kept by pastoralist herdsmen who extensively graze them through the practice of transhumance. Samples from slaughtered cattle at the abattoir were taken from the lower intestinal contents. On the farm, freshly voided faeces were collected. All samples were collected in sterile bijou bottles, placed in a cooler box, taken to the laboratory and cultured within 3 h of collection.

The geographical (GPS) locations for the samples collected and number of positive cases in humans and animals from these locations are attached in Appendix II.

A loopful of each sample was directly streaked onto mCCDA (Blood free *Campylobacter* specific media, modified charcoal cefoperazone deoxycholate agar (Oxoid, UK) containing a selective supplement (SR155E, Oxoid, UK)), immediately on receipt at the laboratory. Plates were incubated in anaerobic jars under micro aerobic conditions of 5% oxygen, 10% carbon dioxide and 85% nitrogen using gas generating packs (Campy-Gen, Oxoid, UK) at 40 °C for 2–4 days. Presumptive colonies were sub-cultured and single colony re-plates made onto blood agar plates.

*Campylobacte*r colony growth was identified as greyish, flat and moist, with a tendency to spread and having a metallic sheen. Single colony re-plates, from presumptive positive colonies from each plate, were stored in cryovials containing nutrient broth with 20% (v/v) glycerol at -80 °C.

### Whole Genome sequencing and assembly

From each of the isolates, genomic DNA was extracted using a Wizard® Genomic DNA Purification Kit (Promega, USA) according to the manufacturers’ protocol. The DNA extracts went through a genome prep similar to Sanger Institutes Illumina Truseq DNA sample preparation protocol (https://support.illumina.com/downloads/truseq_dna_sample_preparation_guide). Briefly, this involved preparing libraries for unidirectional sequencing from 5 µg of genomic DNA from each isolate. Adaptor sequences were added to the ends of the DNA fragments which match the surface bound amplification primers on the flow cells. Sequencing was done on an Illumina HiSeq 2000 analyser with 100 bp paired end runs. Each isolate produced about 400 Mb of 100 bp raw reads which were de novo assembled using the Velvet assembler [15].

### Multilocus sequence typing

Assembled sequences were uploaded into BIGSdb (Bacterial Isolate Genome Sequence Database) at http://pubmlst.org/software/database/bigsdb/ [16]. This software was developed by the University of Oxford and is able to store and analyse sequence information for bacterial species and was used to identify and define loci along the genome from each isolate genome. These identified loci were then organised using an MLST scheme within PubMLST (http://pubmlst.org/campylobacter). Allele number designation to the seven housekeeping loci (*aspA, glnA, gltA, glyA, pgm, tkt, uncA*), allowed assignment of sequence types (STs) to each isolate [17]. Sequences of new alleles for each locus were submitted to the curators for assignment of new allele numbers. Isolates were then separated into STs and based on clonal relationships, grouped into clonal complexes (CC).

### Genome diversity

Genome diversity was investigated using eBURST, Simpson’s Diversity Index, Nei’s Genetic Distance and Rarefaction analysis. Genetic diversity could not be measured or compared for the isolates from cattle as all were the same ST and CC so were excluded from the analysis above except for eBURST.

7-locus allelic profiles of all the isolates in this study were uploaded and analysed in eBURST to determine relatedness and explore diversity within the isolates. The whole input population was analysed as a single group and visualized as the number and sizes of the linked STs in eBURST (http://eburst.mlst.net). The minimum number of identical loci for a group definition was six while the minimum single locus variant (SLV) count for each group definition was three. Bootstrapping with resampling (1000 times) was conducted to provide statistical confidence in ST assignment. The eBURST algorithm subdivides MLST datasets into related groups of STs or CCs and then determines the best arrangement of isolates in the group to the predicted founder [18].

To quantify the genetic diversity within the sets of isolates, Simpson’s Diversity Index was used. This considers the relative frequency of STs in a specific source [19]. A diversity index of 0 indicates that all the isolates are genetically identical while a value of 1 indicates all the isolates are different. To generate confidence intervals, the isolates were bootstrap resampled with replacement 10,000 times using Pop Tools (add-in for Microsoft Excel) allowing the Simpson’s Diversity Index and 95% confidence interval (CI) to be calculated.

To investigate the genetic distance between *Campylobacter* isolates in different pairs of sources, Nei’s genetic distance was used [20]. Nei’s genetic distance compares genetic relatedness between two populations and was calculated using 7-locus MLST data with randomisation and replacement 10,000 times in Microsoft Excel to calculate confidence intervals and P-values. A value of 1 indicates an absence of any similar genotype between the pair while a value of 0 shows that both populations are identical.

To evaluate whether the maximum number of genotypes from a particular animal source had been sampled, rarefaction analysis was employed. Rarefaction is a resampling technique that assesses the proportion of the genotypes from a given source that have been sampled. The results are displayed as a curve which reaches a plateau if all the genotypes have been sampled or the curve keeps increasing where the genotypes in the population remain under sampled [21]. The slope of the curve is also an indication of the strength of diversity within the source with a steep curve indicating a greater diversity than a gradual curve.

### In silico identification of genetic determinants of antimicrobial resistance

Assembled sequences which had been uploaded into BIGSdb, as detailed above, were screened using the autotagger functionality within BIGSdb. Loci were defined by nucleotide sequences with automatic recognition of the query type and the appropriate BLAST algorithm called. This was used to identify loci responsible for fluoroquinolone resistance *gyrA* (PubMLST-Camp 0950), macrolide resistance 23S rRNA (PubMLST-23S rRNA), tetracycline resistance (PubMLST-Camp1698) and beta-lactam resistance (PubMLST-CAMP0265). Resistance was inferred by the presence of one or more alleles at these loci.

### Statistical analysis

Statistical analysis was performed to determine odds ratio (OR), 95% confidence interval and P-values. The OR measures association between the presence or absence of two properties with confidence intervals indicating the spread of variation associated with the value. Comparisons using OR selected one character as the reference. Statistical significance was assumed if the confidence interval for a comparison did not overlap the OR of 1 and P ≤ 0.05.

## Results

### Distribution of *Campylobacter* in humans

From the 50 human isolates recovered, 30 were *C. jejuni* (60%), 17 *C. coli* (34%) and three *C. hyointestinalis* (6%). Only four of the isolates were from patients at health centres while 46 were from asymptomatic carriers in various schools.

The significance of the human characteristics age, gender and clinical manifestation, in relation to *Campylobacter* carriage, was calculated (Table 1). *Campylobacter* was more likely to be isolated from stool samples of asymptomatic individuals in relation to symptomatic ones. Based on age, individuals aged ≥ 15 were significantly less likely to be infected than individuals < 5, with an odds ratio < 1 and P-value < 0.05. Age group 6–14 also had an odds ratio < 1, although this effect only approached significance (P = 0.1). Therefore, isolation of *Campylobacter* tends to decrease with age. Calculations based on gender suggest it is not statistically significant for *Campylobacter* infections.

**Table 1:** *Campylobacter jejuni* and *Campylobacter coli* from human samples by age, health status and gender

	Category	No. Samples	Positive	Negative	OR (95% CI)	P-value
	All	568	47	521		
Age	0 - 5	293	39	253	1	
	6-14	96	7	89	0.5 (0.2, 1.15)	0.1
	≥ 15	179	1	178	0.04 (0.005, 0.26)	0.001
Health Status	Symptomatic	248	4	244	1	
	Asymptomatic	320	43	273	9.6 (3.40,27.16)	< 0.0001
Gender	Male	268	21	247	1	
	Female	300	26	270	1.13(0.62,2.06)	0.7

Odds ratio and p-values have also been calculated

### Distribution of *Campylobacter* in livestock

A total of 312 poultry samples were analysed. Faecal samples were collected and cultured from chicken (layers, broilers, Nigeria indigenous Fulani ecotype) and other poultry (duck, geese, quails). 124 (40%) samples were confirmed positive for *Campylobacter,* and these were identified at different frequencies from all the poultry types. Species specific PCR revealed only *C. jejuni* (25%) and *C. coli* (75%) (Table 2).

**Table 2.** *Campylobacter* spp. isolation from poultry and cattle

	Category	No. Samples	No. Positive Samples		
			*C. jejuni*	*C. coli*	Total (%)
Poultry	Layers	155	6	63	69 (45)
	Broilers	96	7	22	29 (30)
	Indigenous Breeds	47	15	6	21 (45)
	Other	14	3	2	5 (36)
	Total	312	31	93	124 (40)
Cattle	Abattoir	149	0	8	8 (5)
	Farm			11	0
	Total			160	0

Cattle faecal samples were collected from the abattoir (93%) and from a farm (7%). Of the 160 cattle faecal samples analysed, 8 (5%) were positive for *Campylobacter*. Speciation of the isolates through multiplex PCR revealed all of them to be *C*. *coli* (Table 2). All the positive samples were sourced from the abattoir.

### Genomic diversity of *Campylobacter* strains in humans and livestock

**Fig. 1:**
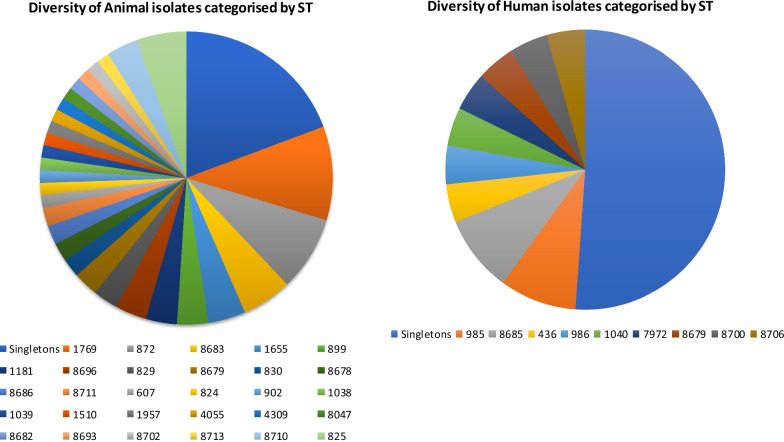
Sequence Type diversity of isolates in samples from animals and humans

A total of 80 STs were identified from 132 *Campylobacter* isolates from animals and 47 isolates from human sources (Fig. 1).

Thirty seven STs were identified from 108 (60%) isolates and had been previously described in PubMLST. The remaining 43 STs from 71 (40%) of the isolates were previously undescribed and these were uploaded into the PubMLST database, curated and assigned new ST numbers (Table 3).

**Table 3:** Novel STs, MLST allelic profiles and host source

Source	aspA	glnA	gltA	glyA	pgm	tkt	uncA	ST (MLST)	clonal_complex (MLST)
Human stool	273	39	516	82	248	43	17	8679	ST-828 complex
Human stool	8	604	5	53	11	3	1	8684	ST-607 complex
Human stool	33	39	30	82	189	678	17	8685	ST-828 complex
Human stool	20	164	24	604	27	19	555	8689	UA
Human stool	63	164	24	188	878	19	557	8690	UA
Human stool	28	34	27	33	223	428	213	8691	UA
Human stool	63	164	24	722	780	19	18	8694	UA
Human stool	63	164	557	21	780	266	18	8695	UA
Human stool	461	34	559	723	879	682	33	8698	UA
Human stool	131	39	150	79	188	534	17	8700	UA
Human stool	1	2	42	4	90	9	8	8703	ST-42 complex
Human stool	103	195	103	140	188	368	79	8704	ST-1150 complex
Human stool	33	38	30	79	880	64	17	8705	UA
Human stool	28	648	27	33	697	61	303	8706	UA
Human stool	33	39	150	174	104	35	17	8707	ST-828 complex
Human stool	1	2	42	4	90	683	8	8708	ST-362 complex
Chicken	33	603	65	82	113	47	17	8677	UA
Chicken	131	38	150	79	805	534	17	8678	UA
Chicken	273	39	516	82	248	43	17	8679	ST-828 complex
Chicken	22	61	4	64	74	3	23	8680	ST-1034 complex
Chicken	22	364	4	64	74	3	23	8681	ST-1034 complex
Chicken	33	39	66	82	104	47	41	8682	ST-828 complex
Chicken	33	39	30	82	678	43	17	8683	ST-828 complex
Chicken	8	604	5	53	11	3	1	8684	ST-607 complex
Chicken	33	39	30	115	104	47	17	8686	ST-828 complex
Chicken	103	110	103	350	188	368	79	8688	ST-1150 complex
Chicken	131	39	30	79	805	534	17	8692	UA
Chicken	435	39	30	79	805	534	17	8693	UA
Chicken	273	39	30	82	248	35	17	8696	ST-828 complex
Chicken	24	30	515	2	89	59	6	8697	ST-460 complex
Chicken	436	39	30	79	805	534	17	8699	UA
Chicken	7	27	1	10	11	3	6	8701	ST-574 complex
Chicken	273	39	30	82	188	35	17	8702	ST-828 complex
Chicken	2	4	1	25	11	358	5	8709	ST-48 complex
Chicken	33	38	150	82	877	43	41	8711	UA
Chicken	103	195	103	140	188	368	17	8713	ST-1150 complex
Chicken	8	2	5	451	11	5	1	8716	ST-607 complex

*UA* unassigned

The five most common STs overall were ST-1769 (n = 15), ST-872 (n = 12), ST-8683 (n = 8), ST-825 (n = 8) and ST-1655 (n = 7). Most of the STs (n = 144) clustered into eighteen different clonal complexes, however, 35 (20%) of the isolates could not be assigned to a CC. This included nineteen isolates from human and sixteen from chicken sources. The most common clonal complex and by far the largest group, was ST-828 which had 99 isolates (55% of the total isolates). This was followed by the ST-607 complex which comprised of 9 isolates (5%). By diversity of STs within the two species, 25 STs were identified from 103 *C. coli* isolates and 30 STs in 76 *C. jejuni* isolates.

Interestingly, nine STs were common to both humans and chicken. These were ST-8679, ST-353, ST-523, ST-607, ST-824, ST-899, ST-1181, ST-1655 and ST-8684. These clustered into five CCs with CC-828 having the highest number of STs (n = 4).

### Population relationship

**Fig. 2:**
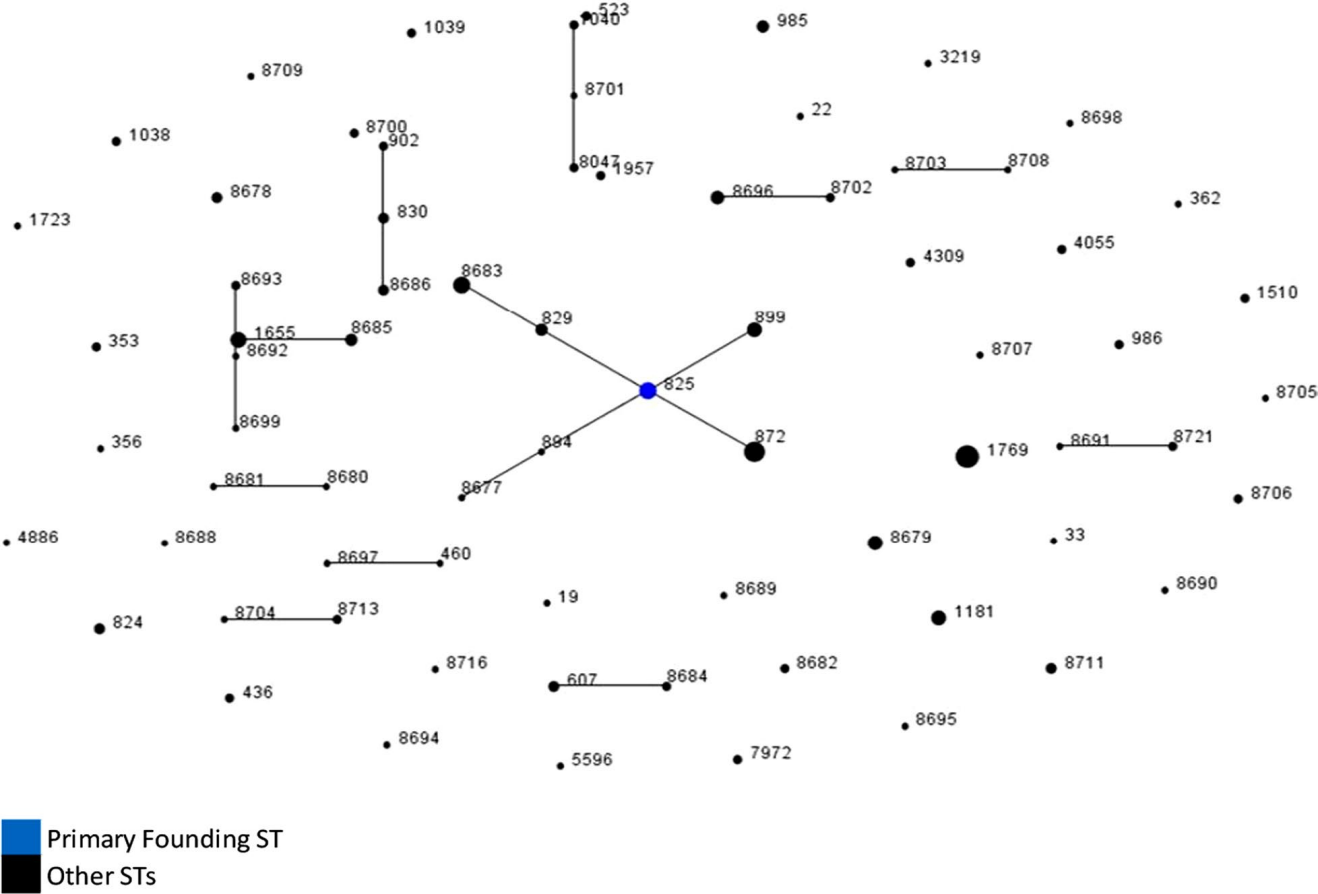
A population snapshot of human, chicken and cattle strains using eBURST. ** STs are indicated by dots which are linked by a line if they are SLVs while the primary founder by a blue dot. Relative abundance of the STs is reflected by the area of the dot

The phylogenetic model eBURST showed relatedness between strains from Nigeria at the 7-locus MLST level and revealed twelve groups of linked STs and 40 singletons. Most of the STs though, were unlinked and were not SLVs of other STs in the population (Fig. 2).

**Fig. 3:**
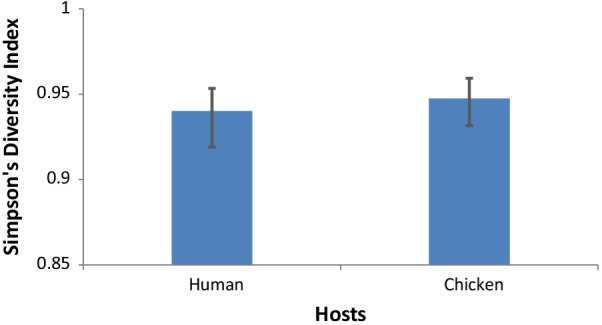
Simpson’s Diversity Index of Campylobacter STs in human and chicken

Simpson’s diversity index was determined using 7-locus MLST data for chicken and human isolates. Diversity was based on the variation in the frequency of individual STs in the total population within each source (Fig. 3).

The diversity index for both human and chicken isolates was only marginally different at 0.940 and 0.947. These values which are close to 1 would suggest a high genetic diversity within the sets of isolates.

**Fig. 4:**
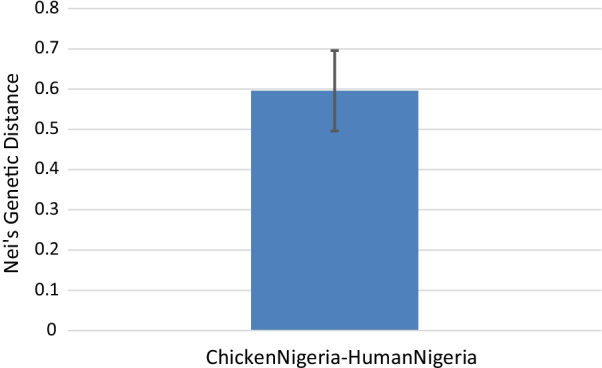
Pairwise Nei’s genetic distance between human and chicken isolates calculated at the 7-locus MLST level

Nei genetic distance between isolates from humans and chickens was calculated at the level of 7-locus MLST where a value of 0 indicates identical genotypes between the pair, and a value of 1 shows an absence of similarity (Fig. 4).

The computed Nei’s genetic distance between chicken and human strains was 0.596 (CI = 0.65, 0.54) and indicates some genetic overlap between human and chicken *Campylobacter* strains.

**Fig. 5:**
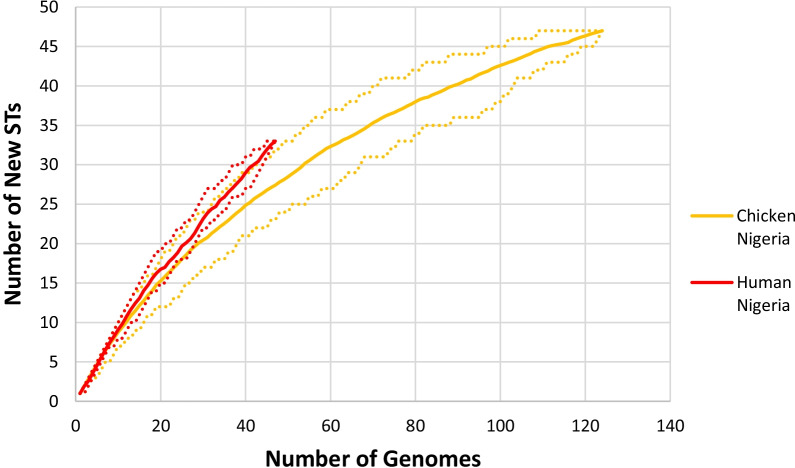
Rarefaction analysis of human and chicken strains. *Dashed lines indicate 95% bootstrapped CI

Diversity within hosts and range of sampling was also assessed by rarefaction analysis using the ST of isolates from humans and chicken (Fig. 5).

Rarefaction curves of ST accumulation in humans and chicken revealed similar steepness in the curves implying that all the sources had very diverse strain sets. None of the rarefaction curves had plateaued indicating that the detection of STs from individual hosts had not yet been saturated. The rarefaction curve in humans though revealed a somewhat higher number of new STs as indicated by the slightly steeper curve.

### Resistance to antibiotics

#### Identification of antibiotic resistance determinants

Resistance determinants for beta-lactam, fluoroquinolone, tetracycline and macrolide classes of antimicrobials in *Campylobacter* are varied and those identified in this study have been listed in Table 4. OXA beta-lactamases, which confer resistance to beta-lactam antimicrobials, were the most frequently occurring resistance determinants and were identified in 68% (122/179) of the *Campylobacter* isolates in this study. Within the group, OXA-193 (49%, n = 87) was the most prevalent OXA beta-lactamase detected while nine other OXA beta-lactamases (n = 35) were detected but at much lower frequencies. Four novel OXA beta-lactams were also detected and have been highlighted in Table 4. Fluoroquinolone resistance was the next most prevalent resistance and was primarily mediated by a mutation at T86I (66%, n = 119). This mutation was the single most frequently occurring resistance determinant in the study. Also observed in the same group was a double mutation, P104S/T86I which occurred in two isolates. For resistance to tetracycline, two determinants were identified. The first, *tet*O, was more common and occurred in 23% (n = 42) of the isolates, followed by the chimeric *tet*(O/32/O) gene which occurred in 1% (n = 2) of the study isolates. Resistance to macrolides was the least occurring, observed in only 2.8% (n = 5) of the isolates and was determined by a change in the 23S rRNA gene, specifically A2075G.

**Table 4:** Antibiotic resistance determinants identified from the study isolates

Betalactams		Fluoroquinolones		Tetracyclines		Macrolides	
Oxacillinase	Frequency (%)	gyrA	Frequency (%)	Tet gene	Frequency (%)	23S rRNA	Frequency (%)
Oxa-193	87 (49)	T86I	119 (66)	tet(O)	42 (23)	A2075G	5 (2.8)
Oxa-449	6 (3)	P104S, T86I	2 (1)	tet(O/32/O)	2 (1)		
Oxa-460	3 (2)						
Oxa-184	2 (1)						
Oxa-61	2 (1)						
Oxa-461	1 (0.6)						
Oxa-783*	9 (5)						
Oxa-784*	9 (5)						
Oxa-785*	2 (1)						
Oxa-786*	1 (0.6)						
Total (%)	122 (68)		121 (67.6)		44 (25)		5 (2.8)

*Previously unidentified OXA beta-lactams

Resistance to all the antibiotics was found in only one isolate, 23 were resistant for up to three antibiotics, 60 to two, 168 to at least one while 24 did not have any resistance characters and were assumed to be susceptible to the four antibiotics. Multi-resistance was observed in only one human isolate while the rest were from chicken.

## Discussion

Human health in developing countries is inseparably associated with the livestock they own with these animals serving as a source of nutrition, labour and extra income. An outcome of this is increased direct contact between humans and livestock or their faeces making it likely for human exposure to zoonotic disease causing pathogens such as *Campylobacter*.

This study showed significant correlation between age group and *Campylobacter* isolation. Children are more likely to be infected with *Campylobacter* because of exposure to faecal contaminated playgrounds [22, 23] and picking items from the ground to put in their mouths. This agrees with age related studies in Nigeria which identified *Campylobacter* to be more common in children [24–26] than adults. A significant association was also determined between *Campylobacter* infection and asymptomatic individuals (OR = 10.5, CI = 3.73, 29.57) who were again mostly children. The likely reasons for the high levels of asymptomatic cases could be increased immunity due to constant exposure to the pathogen through contact. Primary episodes of campylobacteriosis in naïve individuals has also been shown to prevent a subsequent more serious bloody diarrhoea from occurring, and over time the disease totally [27]. However, a study in Malawi comparing the epidemiology of *Campylobacter* infections within the context of a resource poor setting, reported a lower detection rate for *Campylobacter* in non- diarrhoeic children (14%, 71/507) and a higher rate (21%, 415/1941) in children with diarrhoea [28].

The strain diversity both within and between the sources of *Campylobacter* strains in Nigeria revealed a genetically diverse population. A total of 179 isolates were typed with 80 different STs identified. This diversity and relatively high percentage of new STs reflects a heterogenous *Campylobacter* population in Nigeria which also points to the limited number of studies on indigenous *Campylobacter* in Nigeria [26] with only one previous molecular based study uploading MLST sequences into the PubMLST database [29].

The two most frequent human STs were ST-985 and ST-8685 together totalling 17% of the *Campylobacter* population. ST-8685 belongs to CC-828, was unique to this study and could be an ST which has been geographically restricted to Nigeria or has evolved over time to sustain itself within the local environment. On the other hand, ST-985 (CC-403) was not novel to this study and was reported in humans from the Netherlands, Luxembourg, Bangladesh and the UK (pubmlst.org/campylobacter/).

MLST typing of the 124 chicken isolates revealed 47 STs assigned to 16 CCs with 21 STs previously undescribed while 16 STs were unassigned to any CC. Poultry are considered a natural reservoir of *Campylobacter* spp. with studies indicating diverse ST populations in broiler flocks at slaughter [30, 31]. These strains may be circulating within chicken populations or may originate from other sources such as the farm environment, wild animals or other livestock.

A dominance of *C. coli* species (75%) was observed from *Campylobacter* isolates in chicken and which were mostly CC-828 (85%, n = 81). Similar findings where CC-828 was the dominant CC among *C*. *coli* isolates, have been shown [32]. However, the prevalence of *C*. *coli* in chicken is higher in this study than reported elsewhere [33, 34].

The two dominant STs in chicken from this study were ST-1769 (n = 12) and ST-872 (n = 10) both of which are *C. coli*, CC-828 strains. This type of population structure where a few genotypes are predominant within the group seems to occur regularly in chicken flocks [35, 36] and could be due to geography or host specific factors [37]. Isolation of ST-1769 has been previously documented from chicken meat in a study from Croatia [38] and in humans and poultry from other countries like Botswana, USA, Germany and Portugal (https://pubmlst.org/campylobacter/). The over-abundance of ST-1769 strain in chickens in Nigeria could suggest a natural selection and maintenance of phenotypic variations that confer an advantage to survive within its host [39, 40].

A total of 22 *C. jejuni* STs were identified in chicken. None of the STs had a frequency of more than two isolates revealing a high diversity of the *C. jejuni* population in chicken. Only a little evidence exists of a phylogenetic relationship between the numerous clonal complexes in the *C*. *jejuni* population structure [41, 42] making the species highly varied with many STs identified in chicken [43].

In cattle, the 5% incidence of *Campylobacter* isolation in this study is most probably an under estimation of the colonisation in the general cattle population in the state as an earlier study had detected an 18.5% prevalence rate [44]. Also, all the 8 isolates from this source turned out to be from the same ST, ST-825 and CC-828. Previous studies of cattle from Plateau State yielded a greater number of STs and CC’s [29].

Nine *Campylobacter* STs from this study’s’ isolates were found in both human and chicken sources. The ability of *Campylobacter* to colonise animal reservoirs and subsequently infect humans has been shown in STs such as ST-21 which have been described as generalist because they show high genetic flexibility and express diverse fitness factors enabling adaptation in changing host environments [45]. These genetic characteristics has enabled certain livestock specific *Campylobacter* strains to infect and cause disease in humans. Genetic relatedness of *Campylobacter* within the same country between humans and chicken has previously been reported in the UK with the assertion that chicken is the main source for human campylobacteriosis [46, 47].

The extent of diversity from the two main sources, was also seen in the analysis using eBURST. Only one founding genotype, ST-825, was identified within the study population. Most of the other STs were individually unlinked and not single locus variants of the other STs. This level of diversity is possible in instances where high rates of recombination decrease the degree of clonality [48]. Humans also harboured more diversity than chickens as seen by the slope of the curve in the rarefaction analysis and is similar to what was discovered by Gormley et al. (2008) in their analysis of *Campylobacter* in Scotland [49]. Quantifying genetic diversity within both hosts (chickens and humans) indicated a lack of significant difference in the variety of infective strains. This could be attributed to exposure to a large diversity of infecting strains from the environment through foraging for food in the case of chickens or drinking contaminated water and outdoor activities in humans [50–52]. Constant interaction between humans and chicken within the same environment in Nigeria implies that either chickens contribute in part to human infection or both humans and chickens have at least one similar infection source. Nei’s genetic distance supports the assumption that chickens are an important source of infection for humans in Nigeria, however, other sources are likely to also be important. This is also a view shared by other researchers [50].

Antimicrobial resistance in *Campylobacter* isolated from livestock, especially chicken, is of significant public health concern as poultry (indigenous Fulani ecotypes, broilers and layers) are kept by many households at a subsistence or commercial level [53]. Resistance to similar antimicrobials used for human therapy makes it an even more important problem as human contact with faecal droppings from extensively reared chicken, cattle and other livestock increase the risk of infection from these resistant strains and a possible transfer of resistance to humans. Resistance to betalactams, fluoroquinolones and tetracyclines were observed in this study both in livestock and humans with resistance in macrolides found solely in livestock.

Resistance to beta-lactams (42%) and fluoroquinolones (41%) were overrepresented within the study dataset. Beta-lactam resistance is mediated majorly by OXA-61 beta-lactamases and other beta-lactamases [54]. Beta-lactams such as ampicillin are hydrolysed by beta-lactamase enzymes which are widely distributed in *Campylobacter* [55]. This was shown in a UK survey of poultry associated *Campylobacter* where 52% were ampicillin resistant of which 92% possessed bla_OXA-61_ [54]. The high rate of this resistance in Nigeria may be due to widespread use of betalactams such as ampicillin in humans and livestock which has exerted selective pressure on *Campylobacter* to acquire betalactamases [56, 57].

Resistance to fluoroquinolones occurs due to a mutation in the quinolone resistance determining region on *gyrA* [58] and apart from two isolates which had double mutations (T86I and P104S), mutation of *gyrA* was limited to T86I which confirms this as the most common *gyrA* mutation. Other studies in Botswana and Portugal also detected T86I mutation in *gyrA* as the most common type of mutation [59, 60]. It is established that treatment in poultry with fluoroquinolones leads to resistant *Campylobacter* phenotypes [61] and of interest, fluoroquinolone resistant *Campylobacter* do not display a fitness cost and can compete favourably with susceptible *Campylobacter* in the absence of the antibiotic [62].

Tetracycline was the most imported class of antimicrobial to Nigeria in 2014- 2015 [63]. Resistance to this antimicrobial in this study though, was the third most common with an overall detection rate of 23% (44/192) and was lower than what was reported in Spain (72%) [64] and Canada (50%) [65]. Tetracycline resistance is mediated primarily by *tet*O, a ribosomal protection protein transferable as a plasmid encoded gene [66] or is chromosomally located [67]. Other tetracycline resistance genes exist and are able to confer resistance [68]. *Tet*O gene was the most common identified in the study isolates occurring in all the tetracycline resistant isolates except two which had a mosaic derivative *tet*O/32/O gene. Mosaic tetracycline resistance genes have been identified previously occurring in human and animal faecal samples [69] and in other bacteria [70, 71].

Mutations in the 23S rRNA gene which confers resistance to macrolides was observed in only five isolates (2%) and was the least common resistance. Low detection of macrolide resistance has been reported in *Campylobacter* from humans and chicken in Botswana [59] and Poland [72] though higher numbers were detected in an Italian study where the A2075G 23S rRNA mutations were observed in 49 (25%) of *Campylobacter* isolates [73]. Interestingly, it was observed that all the isolates that were resistant to macrolides were also multidrug resistant to tetracyclines and fluoroquinolones.

Multidrug resistance, defined as resistance to three or more antimicrobials [74], was detected in 24 (13%) of the study isolates. Most of the multidrug resistance was to beta-lactam, fluoroquinolone and tetracycline class of antibiotics and may reflect the indiscriminate use of these classes of antibiotics in humans and livestock. A similar finding was observed in China where a study reflecting on the overuse of different antimicrobials in the poultry industry, identified up to 90% prevalence rates of multidrug resistance in *Campylobacter* species [75].

## Conclusion

A thorough understanding of *Campylobacter*, its epidemiology, infection in humans, reservoir status of animal hosts and risk factors for human infection remains limited in the developing world and especially Nigeria. This is because little research has been carried out in this setting to ascertain the pathogenesis and socio-economic burden of the infection.

The use of molecular typing techniques such as MLST and whole genome sequencing to detect genetic traits and characterize isolates in this study proved to be extremely useful in determining the abundant diversity in circulating *Campylobacter* spp. The different genotypes within human and livestock sources in Nigeria was demonstrated and can prove to be very useful in future studies to further understand the pathogen.

Resistance to antimicrobials has been shown both in humans and animals although in humans, treatment for *Campylobacter* enteritis remains controversial except for the immunocompromised and during pregnancy. Severe disease in the undernourished in developing countries though is common and for these group of individuals, options for susceptible oral therapies is diminishing.

In Nigeria, *Campylobacter* infections in humans and animals is poorly understood and more research needs to be done to understand the pathogen. This knowledge would provide links to human infective sources where targeted interventions can be applied to reduce human disease.

## Data Availability

The datasets used and/or analysed during the current study are stored at https://pubmlst.org/organisms/campylobacter-jejunicoli, and can be made available from the corresponding author on reasonable request.

## Appendix

### Appendix II. Geographical relationships between positive cases and sampling locations

**Table:**

		Humans				Animals			
		Total sampled	No. +ve	C. jejuni	C. coli	Total sampled	No. +ve	C. jejuni	C.coli
Jos North									
9° 54' 50.364'' N	School								
8° 53' 22.524'' E	Health Centers	73	0	0	0				
	Markets					149	8	0	8
	Farms								
Jos South									
9° 54' 50.364'' N	School	125	18	9	9				
8° 53' 22.524'' E	Health Centers	194	4	2	2				
	Markets					293	99	12	87
	Farms					42	12	4	8
Pankshin									
9° 19' 42.348'' N	School	42	7	3	4				
9° 25' 17.724'' E	Health Centers								
	Markets					27	13	11	2
	Farms								
Barkin Ladi									
9° 32' 27.204'' N	School	69	11	11	0				
8° 53' 37.788'' E	Health Centers								
	Markets								
	Farms								
Bokkos									
9° 13' 42.727'' N	School	34	5	4	1				
8° 53' 41.288'' E	Health Centers								
	Markets					20	8	4	4
	Farms								

## Abbreviations

bp: Base Pair
CC: Clonal Complex
DNA: Deoxyribonucleic acid
mCCDA: Modified Charcoal Cefoperazone Deoxycholate Agar
MLST: Multilocus Sequence Type
PCR: Polymerase Chain Reaction
rRNA: Ribosomal Ribonucleic acid
SLV: Single Locus Variant
Spp: Species
ST: Sequence Type
